# On the mirror test and the evolutionary origin of self-awareness in vertebrates

**DOI:** 10.1098/rstb.2024.0312

**Published:** 2025-11-13

**Authors:** Masanori Kohda, Shumpei Sogawa, Redouan Bshary

**Affiliations:** ^1^ Department of Biology, Osaka Metropolitan University, Osaka, Osaka Prefecture 558-8585, Japan; ^2^ Department of Biology, University of Neuchâtel, Emile-Argand 11, 2000 Neuchâtel, Switzerland

**Keywords:** mirror self-recognition, photograph self-recognition, self-awareness, false-negatives, shared ancestry hypothesis

## Abstract

Self-awareness in animals is often documented by showing evidence for mirror self-recognition (MSR), which is confirmed by the mirror mark-test. The classic assumption about self-awareness was that it is a complex cognitive process, restricted at best to a few large-brained species. Indeed, early MSR research yielded positive results only in great apes, elephants, dolphins and magpies, while most endotherm species failed. However, recent detailed proof of MSR based on a mental representation of self, i.e. private self-awareness (PrSA), in cleaner wrasse *Labroides dimidiatus*, a small-brained ectotherm fish, indicates that the origin and cognitive complexity of self-awareness must be reconsidered. Here, we first recapitulate key concepts on the evolution of self-awareness, and then summarize the evidence that cleaner wrasse exhibit PrSA. We propose that the many negative MSR results are potentially false-negatives, and that self-awareness does not require a large brain. We posit a new hypothesis: self-awareness was already present in the early shared ancestors of modern vertebrates.

This article is part of the theme issue ‘Evolutionary functions of consciousness’.

## 1. Introduction

Descartes famously assumed that animals lack self-awareness [1]. More recently, the concepts of innate releasing mechanisms (IRM) theory [2,3] and associative learning (classical and operant conditioning) [4,5], which presume that animals do not have self-awareness, have been widely used in psychology to interpret animal behaviours [3]. Although examples of metacognition have recently been reported in some animal species, some argue that those could be explained by associative learning [6]. Thus, specific tests are needed to demonstrate that animals have self-awareness.

For empirical research, demonstrating mirror self-recognition (MSR) with the aid of the mark-test (e.g. [7,8]) is regarded as the crucial method to confirm the existence of animal self-awareness. In this test, subjects are marked with colour on a body part that is not visible to them except when looking at themselves in a mirror. MSR is confirmed if they try to touch or remove the mark on their body only when a mirror is present. Passing this test is difficult to explain by associative learning [9]. Animals touch/scrape the mark smoothly without prior training (reinforcement) and without trial-and-error learning in response to looking at the own mirror-image.

MSR in non-human animals was first experimentally documented in chimpanzees [7]. Since then, MSR tests have been applied to a variety of species (e.g. [10,11]). The initial focus of MSR studies was on its presence/absence to examine the phylogenetic distribution of MSR-capacity. Until recently, such research had yielded positive results only in great apes and a few large-brained endotherm vertebrates, e.g. dolphins [12], elephants [13], magpies [14] and Indian house crows [15]. However, some researchers argued that these studies lack various additional controls in order to exclude alternative explanations [16]. Thus, there is controversy over what results constitute conclusive evidence for MSR [10,16,17]. As recently as 2020, Gallup & Anderson argued that there is no conclusive evidence for MSR in animals except for chimpanzees and to some degree orangutans [16]. In line with this view, anthropocentrism is probably widespread in views on animal self-awareness at present [18–21].

While only a limited number of animal species passed the mark-test, many others failed [7,8,11,22]. These failures have been regarded as evidence that the tested species have no MSR capacity and hence no self-awareness (e.g. [7,8,16,23]). Largely based on this interpretation of MSR-capacity, several hypotheses on the evolution of vertebrates’ self-awareness, including in humans, were proposed. (i) The ‘big bang’ hypothesis: self-awareness evolved once in an ancestor of the Hominid great apes; only they pass the MSR mark-test [7,10,16]. (ii) The ‘gradualist view’ hypothesis: vertebrates have varying levels of self-awareness, ranging from simple to a more complex top level [8,21,24–26]. (iii) The ‘independent evolution’ hypothesis: self-awareness evolved independently in four large-brained clades of endotherms that have ‘mentalizing ability’, i.e. the great apes (including humans), elephants, dolphins and corvids [8,13]. Note that hypotheses (ii) and (iii) are not mutually exclusive. Importantly, discussions on the merits of these hypotheses largely ignore the potential for false-negatives in MSR studies [17,27,28].

There is another discussion on the interpretation of MSR in animals. Morin [29] proposed three kinds of self-awareness in animals, based on [30]: (i) Public self-awareness (PuSA), which is related to visible attributes such as behaviour and physical appearance to which attention is directed [29]. Thus, PuSA treats perceptual self-information. (ii) Private self-awareness (PrSA), which consists of externally unobservable events and characteristics such as emotions, physiological sensations, perceptions, values, goals, motives, etc. In this case, awareness is directed to the animal’s inner life, its mental states. Thus, PrSA involves conceptual self-information, something that is not observable by others. (iii) Meta self-awareness (MSA), where individuals recognize their own PuSA and PrSA [9]. MSA is commonly assumed to be applicable only to linguistic humans [31,32]. Note, however, that Morin [31] insists that speech is also required for the simpler PrSA [31].

A part of the problem about the mark-test is how a positive result should be interpreted with respect to the three levels of self-awareness proposed by Morin [29]. Gallup viewed MSR as a high-level cognitive process, i.e. evidence for PrSA, where its presence would also indicate the presence of theory-of-mind and a concept of death [10]. However, this premise has been criticized, and an alternative possibility that MSR only indicates a form of PuSA, i.e. perceiving the matching of motions between self and the mirror image, has been proposed (e.g. [31,32]). Indeed, the mechanisms of how animals might process the information provided by a mirror had not been studied until very recently [33,34].

**Figure 1.**
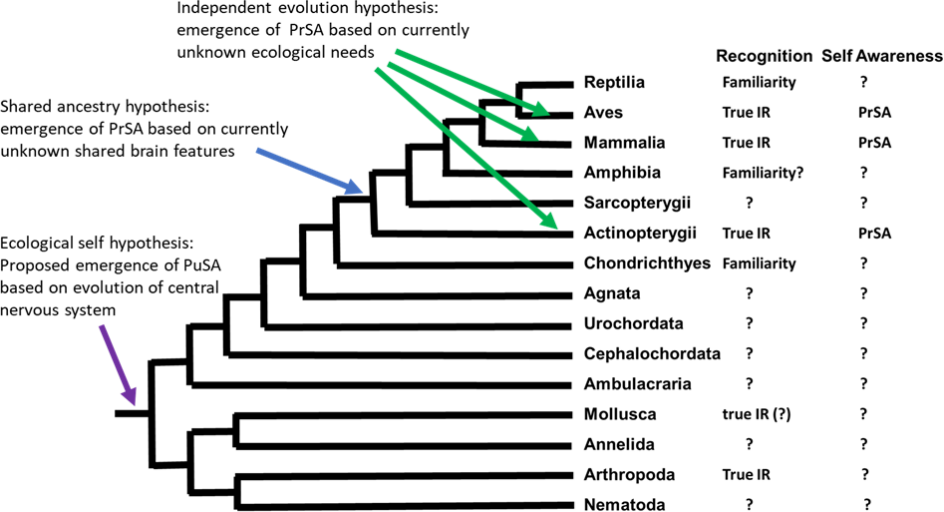
Phylogenetic tree of the major animal clades, giving a summary of the current evidence for true individual recognition (IR) and private self-awareness (PrSA). Arrows indicate where public self-awareness (PuSA) and PrSA supposedly evolved, depending on which hypothesis applies. The tree itself is based on Tudge [37]. Examples of references: familiarity [38] in Reptilia, true IR [39] and PrSA [14,15] in Aves, true IR [40] and PrSA [7,12,13] in Mammalia, familiarity (?) [41] in Amphibia, true IR [42,43] and PrSA [17,33,34] in Actinopterygii, familiality [44] in Chondrichthyes, true IR (?) [45] in Mollusca, true IR [46,47] in Arthropoda.

In our opinion, the most basic adaptive explanation for self-awareness is that any actively mobile species needs to assess the link between its own motor actions and sensory feedback [35], a feat that is visible to anyone and hence has been termed ‘public’. Indeed, human infants show evidence of understanding their own movement long before they pass the mirror test [36]. More generally, Gibson [35] proposed that perception and action entail self-perception or an implicit sense of one’s own body situated and acting in the environment [35]. This view generalizes to all actively moving animals [36]. The ability to move as a distinct entity in space is also called the ‘ecological self’ [36]. If the ecological self is concerned with perceptual self-information, it will be regarded as being equivalent to PuSA. Thus, according to Morin’s definition, accepting the ecological self as evidence for self-awareness (PuSA) would allow us to trace its evolution back to the evolution of central neuronal systems, as found in all bilateria with the evolution of a mesoderm. This would include all clades shown in figure 1, i.e. also nematodes, arthropods, annelids and molluscs. Thus, PuSA could be a very basic cognitive process. In contrast, PrSA would be considerably more complex, as the animal must have a mental representation of itself.

In this article, we will address the various key issues concerning MSR and PrSA. (i) Is the mirror test a reliable test in the sense that negative results indeed indicate the absence of self-awareness? Or is there the possibility that the test produces many false-negatives? (ii) Does a positive MSR test provide conclusive evidence for PrSA, indicating that MSR must be based on a mental representation of the self? (iii) Is PrSA a highly advanced cognitive process that is indicative of other advanced cognitive processes, like theory of mind or metacognition? And (iv) which of the several hypotheses on the evolution of PrSA is best supported by the current data?

Our approach relies heavily on recent mirror test studies on the cleaner wrasse *Labroides dimidiatus*. We first reported MSR in this fish species in 2019 [33], and in several follow-up studies, we identified its underlying mechanisms [17,34,48]. The fact that cleaner wrasses are phylogenetically very distant from the other species known to have MSR capacities makes the data particularly informative for the issues that we address.

## 2. False-negatives in MSR studies

The mark-test has been widely used to test for MSR-capacity in animals. Typically, an animal is regarded as passing the mark test if it touches or scrapes the mark only when seeing itself in a mirror. In contrast, failure to touch the mark is regarded as evidence that subjects do not recognize themselves in the mirror. We argue that such a negative conclusion may often be incorrect.

The history of mark-tests in gorillas provides a prime example of a false-negative [49]. Gorillas failed in passing mark-tests in many studies, except for Koko, who was reared by humans [50]. A proposed explanation is that while ‘normal’ gorillas generally do not gaze at each other’s faces, and hence they also do not look straight at their own mirror image, Koko lacked this social manner and hence passed the test [50]. Several other species also avoid direct eye contact (e.g. rhesus monkey [27]), and hence might also fail the mirror test for that very reason. For these species, there is no equivalent individual to Koko, and hence positive exceptions are lacking. Even in species that show evidence of MSR, i.e. great apes, elephants and magpies, only about 30%–40% of individuals pass the mark-test ([13,14,51], but see [52] for [14]). We consider it to be highly irrational to interpret these results such that within the same species, a passing individual will have PrSA but a failing one does not. Instead, individuals having high curiosity or sensitivity may be more likely to touch the mark, while others would not have the motivation to examine and remove the mark.

An important category of potentially false-negatives concerns the long list of species where individuals do not attempt to remove the mark but are able to use the mirror to gain relevant information (e.g. lesser apes [53]; African grey parrot [54]; pig [55]; sea lion [56]; New Caledonian crow [57]; kea and cockatoo [58]; Eurasian jay [59]; Japanese monkey [60]; capuchin monkey [61]; common marmoset [62]; two *Macaca* monkeys [63], but see [64]). Various colleagues propose that the potential to use mirror reflections as a tool for gaining information does not require recognizing the self in a mirror (e.g. [7,24,54,56]). This is because many species that failed to pass the mark-test can use a mirror as a tool. In contrast, we propose that using a mirror as a tool is an indicator of PrSA. In favour of our hypothesis, we note that individuals that used mirrors as tools forsook being aggressive or at least avoided being distracted by their own mirror image and showed contingency-testing behaviour that may serve to inform whether the mirror reflection is themselves or not. Several species considered by colleagues as not having MSR, such as domestic pigs, rhesus monkeys, manta rays, corvids and domestic fowl, perform contingency-testing behaviours when presented with a mirror and have demonstrated using a mirror as a tool (e.g. [26,27,55,65,66]). We hence hypothesize that the ability to use a mirror as a tool is established after reaching MSR, which is established via contingency-testing of own movements in the mirror but not confirmed by experimenters because subjects do not try to remove a mark.

A simple experiment on cleaner wrasse shows that the risk of producing false-negatives is real if attempts to remove the mark are the sole criterion [17]. Cleaner wrasse were chosen as a suitable fish species to study MSR because of their ecology [9,33]. They have a habit of looking for and picking up small ectoparasites from the bodies of other fish species, so-called ‘clients’ [67,68]. The main ectoparasites of coral reef fishes in the Indo-Pacific are gnathiid isopods, which are 1–2 mm long and look brownish from the distance. Therefore, a brown mark that resembles an ectoparasite should cause recognition of a harmful object and hence cause mirror-trained cleaner fish to try to remove the mark if they have MSR. Indeed, cleaners marked with a small brown colour mark on their throat scraped their throat, but only in the presence of a mirror, and invariably after swimming up to it so that the mark became visible [33]. As long as the mark was brown and the mirror covered an entire aquarium side (yielding permanent exposure to it), almost all cleaners tested so far have passed the mirror test (37/38 tested fish in [17,34]). However, if the mark of the same size of the same material (elastomer plastic) on the same spot was either blue or green, no individual scraped its throat (5 individuals in each colour) [17]. Thus, if the ecological relevance of the mark had not been considered in the original study, cleaner wrasse would have been wrongly classified as lacking MSR.

As far as we know, meaningless marks were used in most studies on the mirror mark-test, and no ecologically relevant marks were used except for with cleaner wrasse. A partial exception involved marking marmosets with a dot of chocolate crème, a highly cherished food, but this still obtained negative results [62]. The marmosets had been well exposed to mirrors before the experiment and they could use the mirror as a tool to retrieve food that was otherwise hidden from view. A potential weakness of the study design is that subjects had not received prior training to eat spots of chocolate crème off visible body parts. However, the authors commented that marmosets tried to eat the spot off the mirror image when seeing their reflection with a chocolate spot. Such a behaviour probably indicates a lack of self-face recognition in this species, although we consider it puzzling that they would approach a stranger to eat from its head without fear of being attacked. We hence acknowledge that it appears to be possible that using a mirror as a tool is a capacity that can be separated from MSR. However, as the cleaner fish example demonstrates that any test using meaningless marks could produce a false-negative [17], mirror mark-tests need to be repeated with ecologically relevant marks in the vast majority of species tested so far.

As a final point of caution against strong interpretation of negative mirror test results, it has been noted that a considerable number of species rely less on vision than on other senses. For example, dogs do not appear to be interested in their mirror image, but they are very much interested in odours. They pass a supposedly olfactory version of the mirror test [69], and wolves may do as well [70]. Yet other species might also have mental representations of individual voices.

## 3. Link between MSR and PrSA

When introducing the mirror test, Gallup proposed that MSR shows that chimpanzees have a mentalistic view, i.e. PrSA [7]. Similarly, de Waal’s ‘self-concept’ [8] and Byrne’s ‘mentalizing ability’ [71] appear to be equivalent to PrSA. In contrast, Morin [29] proposed that in the absence of additional evidence, MSR can in principle also be achieved with the mechanism of kinaesthetic visual matching [29], i.e. matching perceptual self-information with the movements visible in the mirror. Thus, visual matching is a form of PuSA because the movements are visible to third parties [72,73]). As this explanation was not excluded in classic MSR studies, as recently as in 2022, Morin reiterated that there was thus no conclusive evidence for PrSA in animals, including chimpanzees [31].

Humans visually identify multiple known familiar persons by means of a mental image of their faces [74]. Similarly, human self-face recognition in a mirror and on motionless portraits is based on a mental image of self-face, rather than on kinaesthetic visual matching [74]. The use of face-recognition to discriminate between other individuals has been documented in a variety of species. Apart from in primates, non-primate mammals and birds [75–77], this ability has also been reported in relatively small-brained species, including a variety of fishes, e.g. Tanganyika cichlids [78,79], discus fish [80], medaka [81], guppy [82] and stickleback [83], and even in an insect, the paper wasp [46]. These fish and paper wasps live in stable social groups with territoriality, either in a dominance hierarchy or in a sexual pair [46,78,80,83]. The human holistic viewing of a face as evidenced by the ‘face inversion effect’ has been confirmed in some endotherms, as well as in two fish species [81,84]. It has been concluded that subjects that have inner templates of the faces of familiar individuals perform what has been labelled ‘true individual recognition’ (TIR) [42,85]. Importantly for our view on PrSA, it thus appears that the basis for a mental representation of the own face, i.e. the ability to identify others by their facial characteristics, is widespread in animals.

In order to perform an experiment in which positive results would exclude PuSA as a mechanism, cleaner wrasses were exposed to photographs as a test of self-recognition [34]. If animals recognize a photograph of themselves, kinaesthetic visual matching [73,74] fails as an explanation. Thus, these animals must have an inner mental image of the self, i.e. they must have PrSA [34].

After establishing MSR with the standard mark-test, cleaner wrasse did not attack their own photograph but still strongly attacked a photograph of unknown fish (see fig. 3 in [34]). When exposed to digitally composed pictures that showed the four possible combinations between the heads and bodies of self and strangers, subjects did not attack pictures showing their own face but strongly attacked those with a stranger’s face [34]. Furthermore, cleaner wrasses passed the photograph version of the mark-test [34]. In this experiment, unmarked subjects were shown pictures with a brown mark (simulating the presence of an ectoparasite) painted on the throat. The pictures elicited throat scraping only if subjects had prior mirror experience and if the picture was of themselves rather than of a stranger. Taken together, the results present compelling evidence that cleaner fish identify the motionless self-pictures as themselves. Therefore, they must have a mental image of their own face, and thus have PrSA [34].

## 4. Is PrSA an advanced cognitive process indicative of the development of other such processes?

In his classic paper, Gallup restricted his interpretation of the data to chimpanzees having self-recognition, while macaques apparently lack this capacity [7]. Only later did he propose that not only does the MSR provide direct evidence for PrS, but as a consequence it allows one to infer a suite of other supposedly highly advanced cognitive processes. He offered three discernable components to PrSA, which he called senses of continuity, of personal agency and of identity ([86] relating to [87]). The first sense relates to the ability to represent oneself in relation to past, present and future events [88], i.e. the capacity for mental time travel [89]. Personal agency means that being aware of one’s own actions, knowledge and intentions also enables the attribution of mental-states to others, i.e. having a theory of mind [90]. The possession of an identity implies the ability to be aware of one’s own existence and to reflect on death [91]. In addition, there is still the purported link between PrSA and metacognition.

The experiments on cleaner fish should make it clear that all such hypotheses need detailed testing. We propose that PrSA is a more basic cognitive process that may be a necessary prerequisite to achieving mental time travelling, theory-of-mind, a concept of death and metacognition rather than a process that only manifests when the other processes have already evolved. In other words, we propose that if we can observe these processes in some animals then we can infer that they have PrSA, while the inverse inference is not possible. This being said, there is some evidence that cleaner fish possess some basic episodic-like memory [92] and some basic perspective-taking abilities [93].

## 5. What hypothesis regarding the evolution of PrSA is best supported by current data?

From the summary of the current evidence on PrSA, it should be clear that the Big Bang hypothesis is not supported. As cleaner fish have a mental image of self, it is clear that not even a large endotherm brain with its organizational specializations is necessary for PrSA to evolve. Furthermore, we consider the gradualist hypothesis at least unlikely to explain the mirror-test data on endotherm vertebrates. de Waal [8] summarized the gradualist view based on four observations [8]: (i) several species other than the great apes pass the mark test, (ii) some species pass the mark test via multimodal perception with tactile perception, (iii) some species can use the mirror as a tool to look for resources such as food, and (iv) some species that do not pass mark-test can recognize the own mirror image as an uncanny conspecific rather than a real, strange individual. He assumed a stepwise evolution of PrSA from simple to complex, related to performance in MSR tests (fig. 3B in [8]). Various researchers have expressed their agreement with this view (e.g. [21,25,26]). As we see it, the greatest weakness of the gradualist hypothesis is that there is no ecologically motivated hypothesis to explain why a few species pass the mirror test while so many others apparently fail. Until recently, brain size could be used to some degree as a predictor, although positive data in magpies but negative results in ravens and parrots remained difficult to explain. With cleaner wrasse passing the test based on a mental image of self, there is neither an ecological hypothesis nor a brain-size hypothesis that fits the data. Instead, the cleaner wrasse studies suggest that negative results in mirror tests cannot be taken as evidence for the absence of PrSA [17]. More specifically, it seems difficult to explain why a small-brained fish should have evolved a higher level of self-awareness than the majority of endotherms.

The current data are in principle in line with the hypothesis that PrSA evolved repeatedly and independently in various clades. Still, this hypothesis raises important issues. Foremost, there is the aforementioned problem that we lack any ecologically based hypothesis that could explain such repeated independent evolution. One possibility is that two factors may act independently or in interaction with each other: brain complexity and a complex social life. The former may explain why orangutans show MSR despite living a rather solitary life. The latter may explain why cleaner wrasse have MSR despite having an average-sized fish brain for their body weight. Cleaner wrasse are protogynous hermaphrodites, starting their reproductive career as female and only the largest individuals change sex and become males. They thus live in haremic polygyny with territoriality and a size-based dominance hierarchy [67,94,95]. Furthermore, cleaner wrasse have up to 3000 interactions per day with so-called client reef fishes [96], which visit to have ectoparasites removed [97]. These interactions contain both cooperation and conflict, as cleaner wrasse prefer to eat their clients’ protective mucus rather than their ectoparasites [98]. Because of this conflict, cleaners need to assess client strategies in order to optimize their own food intake by adjusting the frequency of mucus feeding acts. Cleaner wrasse respond both to the clients’ willingness to exert choice between potential cleaners and to observers attributing a reputation score to cleaners based on how they treat their current client, with both factors varying as a function of fish densities [99,100]. While there is experimental evidence only for cleaners being able to distinguish between familiar and unfamiliar clients [101], the fact that cleaners respond to being chased by a client after feeding on mucus by providing a better service during the follow-up interaction minutes later [102] can only be explained if cleaners recognize individual clients. Taken together, cleaners appear to perceive agency in individual clients, which may in turn enhance the perception of their own agency. Note that we distinguish between the perception of agency and the attribution of goals and beliefs, i.e. a proper theory-of-mind.

If the complexity of the social life plays a crucial role in the evolution of PrSA, then other social fish emerge as suitable candidates for further testing. Sex-changing haremic species are common in fishes, although complex cleaning interactions are restricted to the genus *Labroides*. Cichlids of Lake Tanganyika are well known for cooperative breeding [103], and strong pair bonds in long-term monogamous relationships may lead to selective prosocial preferences in the convict cichlid *Amatitlania nigrofasciata* [104].

While social complexity is seen as a major selective force on brain evolution [105,106], it is currently unclear whether social complexity is indeed a factor selecting for PrSA. Even in species with a solitary life-style, individuals need to interact with neighbours and find a mate, leading to both competitive and cooperative social interactions. Furthermore, predators and prey may be seen as agents. In this scenario, PrSA may emerge as long as some minimal brain complexity has evolved. Based on this view, we propose an additional hypothesis based on shared ancestry. In this scenario, the current evidence for MSR, most likely based on PrSA, includes teleost fish as well as birds and mammals. Assuming shared ancestry, the current evidence would imply that their shared ancestor already potentially possessed the relevant brain organization for PrSA. Such a shared ancestor would have been a fish species that lived before the split between the Sarcopterygii (the ancestors of tetrapods, which include lungfish and the coelacanth) and Actinopterygii (the ancestors of modern teleost fish) [107]. The implications of the ‘Shared ancestry hypothesis’ for the evolution of PrSA as well as for the ecological self-concept are summarized in figure 1.

## 6. Conclusions/outlook

We suggest that the experiments on cleaner wrasse have fundamentally changed our view on MSR and PrSA. We hope that these experiments and our review inspire the re-testing of species that have hitherto failed in MSR, and the addition of new species that may help to expand our understanding of MSR and of PrSA. Of particular interest could be more research on sharks and rays, but also on selected invertebrates. Cephalopods in particular have large brains for invertebrates, and questions about their minds are prevalent in the literature [108]. Among insects, paper wasps are particularly suitable for MSR studies as they have social hierarchies based on individual face recognition [46,109].

It might be difficult for many colleagues to accept the idea that small-brained fish have such a ‘sophisticated’ ability, and that vertebrates from fish to humans may have PrSA based on shared ancestry. We argue that acceptance will only be difficult if there is confusion between PrSA and a variety of additional cognitive skills. Existence of PrSA means that the animals can understand causal relationships between things and have mental states. It is only the basis for the evolution of a variety of skills such as MSR, true individual recognition, theory of mind, the use of mirrors as a tool, mental time travel and so on, rather than evidence for such skills. Brain sizes will relate to the sum of skills, including perception, motor control and sensory–motor integration [9,110] but not to PrSA itself, as revealed in our studies on cleaner wrasse [34,48].

Many philosophical and scientific theories have assumed that animals lack self-awareness, and that animal behaviour reflects only IRM [2,3] and/or classical and operant conditioning [5]. As discussed in this article, finding PrSA in fish demands a major re-evaluation of such views.

## Data Availability

This article has no additional data.
